# Shear Stress and Microbubble‐Mediated Modulation of Endothelial Cell Immunobiology

**DOI:** 10.1002/smsc.202400489

**Published:** 2025-01-27

**Authors:** Elahe Memari, Davindra Singh, Ryan Alkins, Brandon Helfield

**Affiliations:** ^1^ Department of Physics Concordia University 7141 Sherbrooke St. W Montreal QC H4B 1R6 Canada; ^2^ Department of Biology Concordia University 7141 Sherbrooke St. W Montreal QC H4B 1R6 Canada; ^3^ Centre for Neuroscience Studies Queen's University Botterell Hall, 18 Stuart Street Kingston ON K7L 3N6 Canada; ^4^ Division of Neurosurgery, Department of Surgery Kingston Health Sciences Centre Queen's University Botterell Hall, 18 Stuart Street Kingston ON K7L 3N6 Canada

**Keywords:** acoustics, cellular immunotherapy, focused ultrasound, ICAM‐1, secretomes

## Abstract

Cellular immunotherapy remains hindered in the context of solid tumors due to the immunosuppressive microenvironment, in which key endothelial cell adhesion molecules (CAM) are suppressed. Microbubble‐mediated focused ultrasound is being explored for targeted immunotherapy and can exert local shear stress upon neighboring endothelial cells. However, fluid and microbubble‐induced shear modulation of endothelial immunobiology is not well understood. Herein, the influence of both types of shear stress on human endothelial vein (HUVEC) and brain endothelial (HBEC‐5i) CAM expression and secretion of over 90 cytokines using acoustically coupled microscopy is examined. Fluid flow results in time‐dependent modulation of CAM expression, where ICAM‐1 peaked at 4 h (1.98‐fold, *p* < 0.001, HUVEC) and 24 h (1.56‐fold, *p* < 0.001, HBEC‐5i). While some chemokines are significantly enhanced (up to 16.2‐fold; *p* < 0.001) from both endothelial cell types (e.g., IL‐8, MCP‐1, MCP‐3), others are differentially expressed (e.g., CCL5, CXCL‐16, SDF‐1). Under ultrasound, ICAM‐1 expression at 4 h increased (≈1.4‐fold, *p* < 0.01) and resulted in significant large‐magnitude (*p* < 0.05) differential expression of 20 cytokines, most of which have immune‐activating function and within a subset of those induced by shear‐flow. Microbubble‐mediated ultrasound regulates ICAM‐1 expression and the human endothelial secretome toward an immune cell recruitment paradigm, and thus may reinforce solid tumor cellular immunotherapy efforts.

## Graphical abstract

The article explores how ultrasound and microbubbles under flow conditions are able to modulate the immunobiology of brain endothelial cells; including surface expression of key adhesion molecules and cytokine/chemokine release. Taken together, this highlights the potential for microbubble‐assisted cellular immunotherapy techniques.

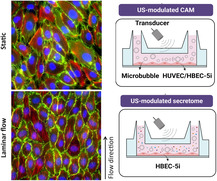

## 1 Introduction

In recent years, immunotherapy has had major successes in the field of oncology—techniques of which include monoclonal antibody therapy (e.g., rituximab targeted against CD20) and more recently immune checkpoint blockade (e.g., pembrolizumab targeted for PD‐1). In addition to antibody‐based strategies, a subset of immunotherapy in which immune cells are either expanded (adoptive cell transfers) or engineered (chimeric‐antigen‐receptor; CAR) ex vivo to specifically express a given T‐cell receptor with hyperaffinity—cellular immunotherapy—has been recently deployed. There have been over 1000 clinical trials worldwide investigating the use of CAR T cells, mostly in lymphoma or leukemia applications using CD19‐specific CARs.^[^ ^1^, ^2^ ^]^ Indeed, this relatively new treatment paradigm has had extraordinary results in blood‐borne cancers; for example, the results from the ELIANA trial showed an overall remission rate of 81% at 3 months^[^ ^3^ ^]^ in patients with relapsed or refractory acute lymphoblastic leukemia and no other treatment options, with a median event‐free survival of 24 months.^[^ ^4^ ^]^ Despite this, cellular immunotherapy approaches have had limited success when targeting solid tumors (e.g., glioblastoma, renal cell carcinoma, colorectal cancer^[^ ^5^ ^]^). This can be attributed to numerous physical and biological immunosuppressive factors including antigen heterogeneity, the downregulation of tumor‐specific endothelial cell surface expression of adhesion molecules (for instance, ICAM‐1, MadCAM‐1), the creation of immunologically inaccessible sites (physical barriers like collagen and fibrin prevent the entry of immune cells), and tumor‐induced immune suppression within the microenvironment (e.g., release of factors that directly inhibit T cells or induce regulatory T cells).^[^ ^6^, ^7^, ^8^ ^]^ Further, solid tumor mechanopathology factors (e.g., increased tumor stiffness)^[^ ^9^ ^]^ can cause extensive vascular compression and heterogeneous blood flow and shear stress profiles, further impeding the success of cellular immunotherapeutic approaches.^[^ ^10^ ^]^ In this context, shear stress plays a critical role in modulating endothelial cell immunobiology within the tumor microenvironment (TME). Shear force sensed by the endothelial cell mechanosensory molecules (e.g., cytoskeleton, integrins, growth factor receptors, ion channels, and several cell adhesion molecules (CAM)) influences the signaling networks involved in inflammatory responses.^[^ ^11^ ^]^ Therefore, shear‐mediated mechanisms that can modulate the suppressive nature of the TME hold a significant potential for improving cellular immunotherapy.

Focused ultrasound (US) is an innovative therapeutic strategy capable of exerting various bioeffects through both thermal and mechanical mechanisms. In hyperthermia, a regime characterized by a mild temperature rise (≈up to 43 °C), an immune response can be elicited by releasing cell debris and activating antigen‐presenting cells,^[^ ^12^ ^]^ and can augment chemotherapy and radiotherapy approaches.^[^ ^13^ ^]^ At higher acoustic intensities, the nonlinear effects of US can increase the temperature beyond 60 °C, known as thermoablation. This technique, capable of causing rapid tissue necrosis, is currently approved for the treatment of essential tremor and tremor‐dominant Parkinson's disease.^[^ ^13^ ^]^ In addition to thermal effects, another mechanism is via mechanical effects which can be exploited with or without clinically used contrast agent microbubbles. An emerging strategy is utilizing a combination of low‐intensity ultrasound with microbubbles, which can locally and reversibly modulate the permeability and gene expression of the neighboring vasculature and surrounding tissue.^[^ ^14^, ^15^ ^]^ The most clinically advanced application of the latter is in the temporary opening of the blood‐brain‐barrier,^[^ ^16^ ^]^ which has been examined as a targeted drug delivery approach in the contexts of neurodegenerative disease (Alzheimer's,^[^ ^17^ ^]^ Amyotrophic lateral sclerosis (ALS),^[^ ^18^ ^]^ Parkinson's^[^ ^19^ ^]^) and neuro‐oncology.^[^ ^20^, ^21^, ^22^ ^]^ Indeed, the delivery of immunomodulating agents to brain tumors using this method, including monoclonal antibodies,^[^ ^23^ ^]^ has reached the clinical trial stage, with many other types of immune‐altering agents currently under preclinical investigations.^[^ ^24^ ^]^

Aside from the targeted delivery of a therapeutic, ultrasound treatment has been shown to modulate intracellular signaling,^[^ ^25^ ^]^ including the release and expression of proinflammatory factors.^[^ ^26^, ^27^ ^]^ Indeed, on a single‐cell level, ultrasound‐stimulated microbubble response has been shown to exhibit physical forces (e.g., shear) on neighboring endothelial cells,^[^ ^28^ ^]^ to cause reversible alterations in both plasma membrane permeability^[^ ^29^, ^30^, ^31^ ^]^ and vascular permeability,^[^ ^32^, ^33^ ^]^ and to initiate sustained calcium ion entry and downstream signaling.^[^ ^34^, ^35^, ^36^, ^37^ ^]^

Given the heterogeneous TME, including the physiological hostile milieu and abnormal shear stress patterns exerted on endothelial cells, our objective here is to ascertain the extent to which microbubble‐assisted ultrasound treatment alters endothelial cell immunobiology and to make qualitative connections to flow‐induced shear stress, with a view toward enhancing immune cell trafficking and recruitment. Using two human endothelial cell lines of differing origins (umbilical vein HUVEC; brain microvasculature HBEC‐5i), we first examined the shear flow‐induced changes in key immune‐cell recruitment surface molecules ICAM‐1 and MadCAM‐1 and the endothelial secretome in a time‐dependent manner. Next, using acoustically coupled microscopy, we explore the ultrasound conditions that, when used with perfusing microbubbles, modulate these same endothelial markers and observe how these secreted factors change as compared to untreated, sham controls. Finally, we conclude with implications of our results in the broad context of ultrasound‐assisted immunomodulation.

## 2 Results and Discussion

### 2.1 The Influence of Shear Flow Preconditioning on Cell Adhesion Molecule Expression

**Figure 1:**
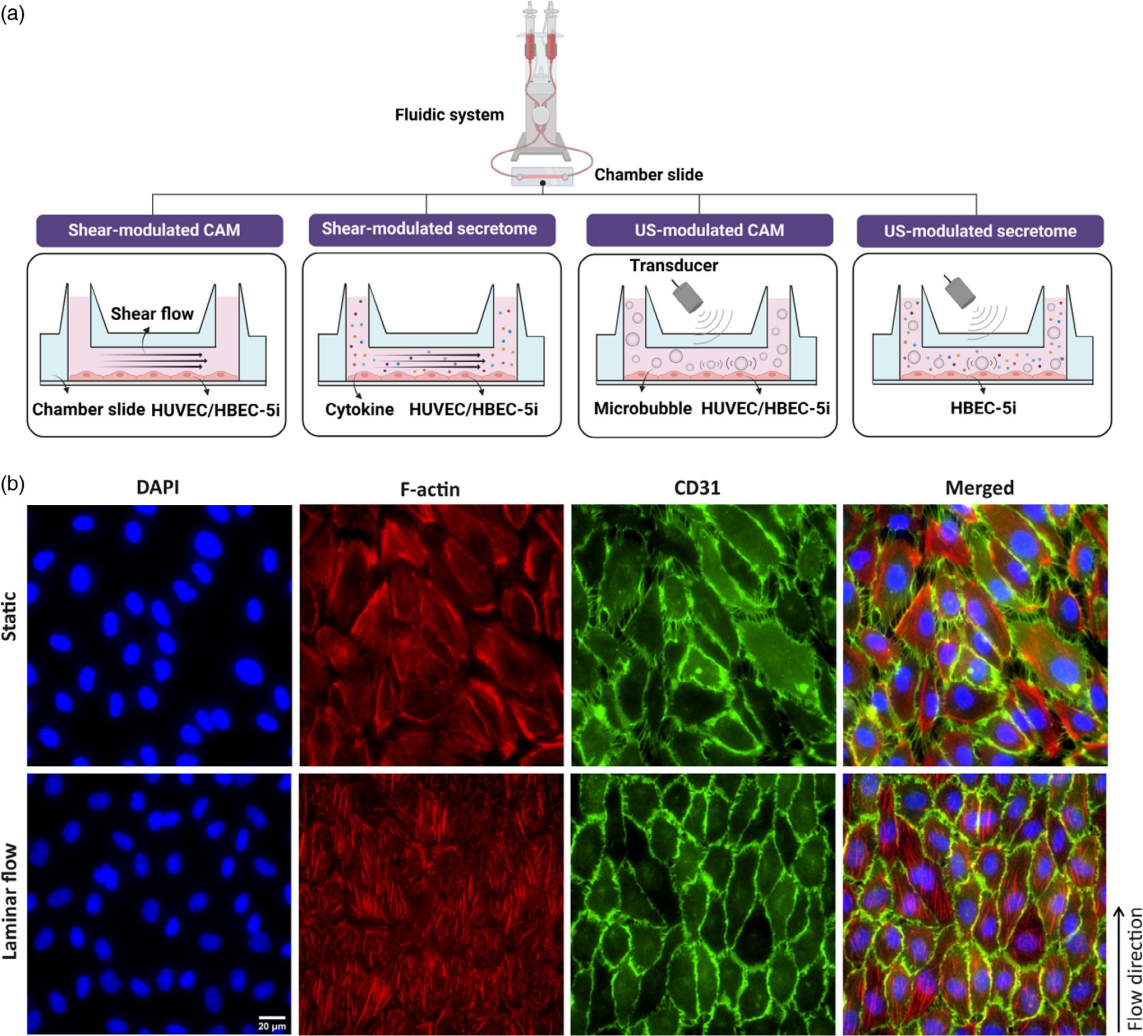
Schematic illustration of the experimental setup and the influence of shear flow on cellular morphology. a) Human endothelial cells, either HUVEC or HBEC‐5i, were subjected to 9 dyn cm^−2^ shear flow for 0–48 h. The effect of laminar shear stress on the surface expression of ICAM‐1 and MadCAM‐1 was conducted using immunofluorescence microscopy, and endothelial secretome profiling was assessed through a multiplex immunoassay. Additionally, the effect of ultrasound‐stimulated microbubbles (1 MHz, 150–210 kPa, 20‐cycle burst, 1 ms PRI, 1–4 min durations) on the surface expression of ICAM‐1 and MadCAM‐1 in HUVEC and HBEC‐5i was investigated. Finally, microbubble‐mediated modulation of cytokine profiles was further studied in HBEC‐5i. b) Fluorescence microscopy images representing the effect of laminar shear flow preconditioning on HUVEC morphology as compared to statically cultivated cells. Cell nuclei are stained in blue, actin filaments are stained in red, and PECAM‐1 (CD31) is stained in green. The fluorescence images indicate that long‐term laminar flow preconditioning results in a more uniform morphology, improved cell‐to‐cell contact, reduced extracellular gaps, and cell orientation in the direction of flow. In contrast, statically seeded cells display highly heterogeneous morphology, random orientation, and significantly larger extracellular gaps between endothelial cells.

**Figure 2:**
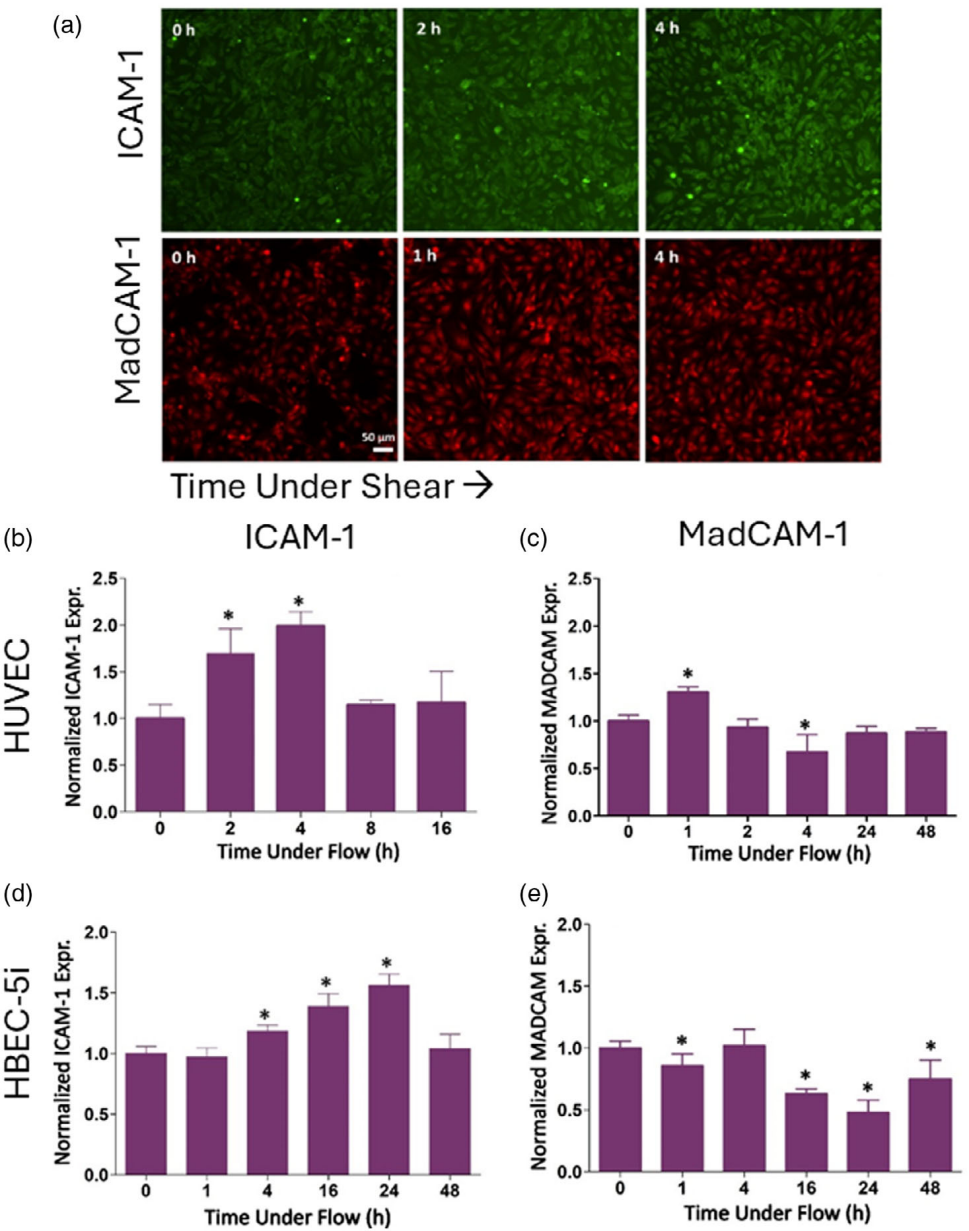
Time‐dependent shear flow differentially modulates the surface expression of ICAM‐1 and MadCAM‐1 on human endothelial cells. A confluent endothelial monolayer was seeded in chamber slides and exposed to 9 dyn cm^−2^ shear flow ranging from 0 to 48 h. a) Representative fluorescence microscopy images illustrating a temporal regulation of ICAM‐1 (top panel) and MadCAM‐1 (bottom panel) expression under shear flow conditions for HUVEC. ICAM‐1 is marked in green, stained by Alexa fluor 488‐conjugated antibody, whereas MadCAM‐1 is shown in red, stained by Alexa fluor 555‐conjugated antibody. For HUVEC, b) quantification of ICAM‐1 expression showed a significant upregulation in response to shear flow preconditioning, with a peak increase at 4 h and returning to baseline by 8 h. c) MadCAM‐1 expression revealed a different temporal pattern, reaching a minimum at 4 h after exposure to shear flow. For HBEC‐5i, d) quantification of ICAM‐1 surface expression revealed a time‐dependent upregulation, with a peak increase at 24 h postexposure to shear flow and it returned to the baseline by 48 h. e) In contrast, MadCAM‐1 expression under shear flow preconditioning showed an opposite trend, and the expression level reached a minimum by 24 h. No statistical difference between the treatment and sham control was observed unless marked on the plot.

As depicted in **Figure** **1**, we first investigated how endothelial cell cultivation under shear flow affects cellular morphology. Shear flow preconditioning led to a more physiologically relevant morphology, characterized with cell elongation in the direction of flow and well‐defined cell‐to‐cell contact. In contrast, statically cultivated cells exhibited a heterogeneous morphology and large extracellular gaps between cells with random orientation (Figure 1b). Then, we examined the extent to which shear flow preconditioning influences the expression of CAM on HUVECs, a more commonly studied human endothelial line of venous origin (**Figure** **2**a–c). Figure 2a depicts representative micrographs of ICAM‐1 (green) and MadCAM‐1 (red) expression at different time‐points of ongoing shear. Quantification of these expression profiles revealed maximum expression of ICAM‐1 as early as 4 h after flow exposure (1.98 ± 0.15‐fold increase compared to no flow, *p* < 0.001), a transient short‐lived effect as evidenced by its decrease back to baseline levels by 8 h (Figure 2b). The shear stress‐induced surface expression of ICAM‐1—a major regulator of leukocyte trafficking to inflammatory sites—has long been known to exhibit time dependence. Indeed, the data presented here is consistent with previous works in which an approximate twofold increase was observed between 4 and 6 h and returned to baseline by >8 h.^[^ ^38^, ^39^ ^]^ The MadCAM‐1 profile (Figure 2c), however, responded quite differently to the flow shear stress, locally peaking slightly after 1 h and then reaching a global minimum by 4 h (0.67 ± 0.18, fold compared to no flow *p* < 0.05). MadCAM‐1 expression has been observed on the brain endothelium in chronically inflamed cerebral vasculature associated with autoimmune encephalomyelitis. It has been suggested that MadCAM‐1 is involved in the homing of α4β7‐integrin expressing lymphocytes.^[^ ^40^ ^]^ While to our knowledge there is no direct information on the effect of shear flow on MadCAM‐1 expression profile, we can perhaps intuit an understanding by examining VCAM‐1, which shares a similar pattern to shear flow preconditioning. It has been shown that under laminar shear stress, VCAM‐1 significantly downregulates in a time‐dependent manner, leading to a reduced number of lymphocytes adhering to the endothelial cells.^[^ ^41^ ^]^

Next, we conducted the same experiments examining shear stress preconditioning and surface expression of ICAM‐1 and MadCAM‐1 on HBEC‐5i (Figure 2d,e). Similar to HUVECs, quantification of microscopy images revealed a time‐dependent upregulation of ICAM‐1 on HBEC‐5i (Figure 2d), however, with a peak increase in expression observed at 24 h (1.56‐fold compared to static, *p* < 0.001) returning to baseline levels by 48 h—resulting in a relatively delayed response compared to HUVECs (Figure 2b). A similar comparative effect is shown with MadCAM‐1 (Figure 2e), in which the surface expression exhibited by HBEC‐5i reached a minimum much later than HUVEC, here at 24 h (0.48 ± 0.10 compared to static, *p* < 0.001). While, to the best of our knowledge, time‐dependent shear flow CAM surface expression of human brain endothelial cells has never been reported, our dataset is broadly consistent with previous work by Cucullo et al.^[^ ^42^ ^]^ in which they demonstrated a small flow‐mediated increase (≈1.25‐fold) in both ICAM‐1 and MadCAM‐1 gene expression in human brain endothelial cells, albeit under an entirely different flow regime. Further, work by Rochfort and Cummins^[^ ^43^ ^]^ demonstrated the time‐dependent shear‐induced (8 dyn cm^−2^) expression of thrombomodulin in human brain endothelial cells, peaking at 24 h—similar in timescale to what we report here for ICAM‐1.

It is not entirely surprising that the temporal nature of shear‐induced CAM expression differs between the human brain microvascular cells (HBEC‐5i) and umbilical vein endothelial cells (HUVECs). It has been long demonstrated that both native levels and proinflammatory cytokine‐induced expression of ICAM‐1 are amplified in HUVEC compared to human brain endothelial cells,^[^ ^44^ ^]^ and a subset of this data confirms drastic differences in static CAM surface expressions across human endothelial cell types of distinct origins.^[^ ^41^, ^45^, ^46^ ^]^

### 2.2 The Effect of Shear Flow Preconditioning on Endothelial Cell Secretome

**Figure 3:**
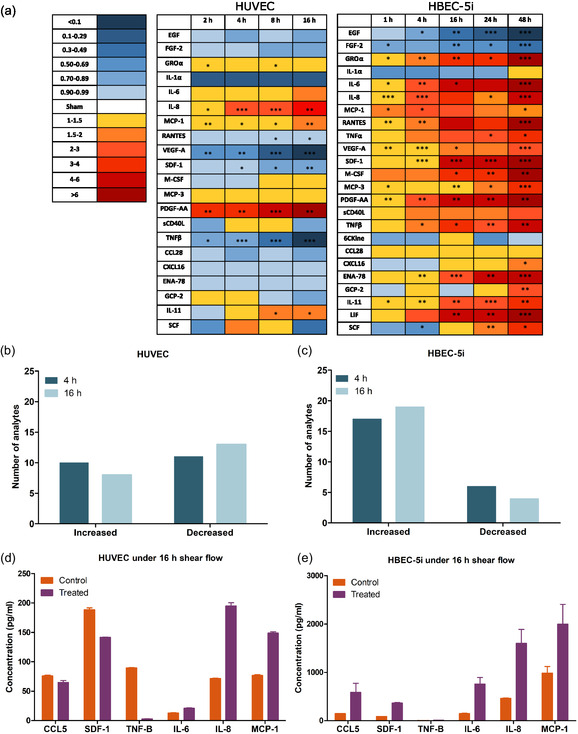
Shear flow preconditioning modulates endothelial secretome based on cell origin. a) Heat maps display the influence of shear flow preconditioning on (left) HUVEC and (right) HBEC‐5i secretomes. The secretion level of different analytes was quantified using Luminex 200 technology and normalized to the static control. Asterisks indicate statistical significance, with *, **, *** denoting *p* < 0.05, *p* < 0.01, *p* < 0.001, respectively. Our results revealed that shear flow preconditioning differently influences both secretomes; shear flow increased the secretion level of most examined analytes in HBEC‐5i, including a subset of immune cell trafficking markers such as CCL‐5, CXCL‐15, and SDF‐1, whereas the secretion level of these factors decreased in HUVEC under similar flow conditions. b,c) A summary of cytokine fold‐change following 4 and 16 h shear flow preconditioning. These plots display the number of cytokines, chemokines, and growth factors that were either upregulated or downregulated in HUVEC or HBEC‐5i in response to 4 and 16 h laminar shear flow preconditioning. Finally, selected factors that were differentially secreted by d) HUVEC and e) HBEC‐5i after shear preconditioning for 16 h are illustrated here. Our findings indicate that a subset of critical factors involved in inflammation and immune cell recruitment exhibit differential responses to the same shear flow conditions depending on the cell origin. Additionally, the baseline concentration of these cytokines varies considerably between these two cell types, with HBEC‐5i generally displaying a significantly higher concentration of proinflammatory mediators than HUVEC.

Given the heterogeneity in flow‐induced shear stress within the TME and its relation to disease progression (e.g., glioblastoma^[^ ^47^, ^48^ ^]^), we investigated the secretome of these cells under the same shear flow condition as above to gain deeper insight into the physiological influence of shear flow preconditioning on endothelial cell immunobiology. Further, we assayed the endothelial secretome as a function of time at the same time points as determined from our surface ICAM‐1 expression dataset. In **Figure** **3**a, the fold‐change in shear‐induced cytokine expression compared to static control as a function of time is displayed for both HUVEC and HBEC‐5i, in which the color is indicative of the magnitude of change (blue is a decrease; red is an increase) and star symbols denote statistical significance. Here, only analytes that exhibited significant signal above the noise floor are shown. With respect to the HUVEC secretome, we observed a significant decrease in the secretion level of a subset of factors such as stromal cell‐derived factor (SDF‐1), CXCL‐16, CCL5, epidermal growth factor (EGF), fibroblast growth factor‐2 (FGF‐2), IL‐1α, vascular endothelial growth factor‐A (VEGF‐A), tumor necrosis factor beta (TNF‐β), CCL28, and epithelial‐neutrophil activating peptide 78 (ENA‐78). The observed results for HUVEC align with previous studies, reporting that high laminar shear stress under short durations suppresses the signaling pathways involved in the proinflammatory responses.^[^ ^49^, ^50^ ^]^ As displayed in Figure 3a, there is a stark contrast when considering the HBEC‐5i secretome, which revealed a significant increase in the concentration of 19 cytokines, chemokines, and growth factors including GRO‐α, IL‐6, IL‐8, MCP‐1, RANTES, TNF‐α, VEGF‐A, SDF‐1, M‐CSF, PDGF‐AA, sCD40L, TNF‐β, CCL28, CXCL16, ENA‐78, GCP‐2, IL‐11, leukemia inhibitory factor (LIF), and SCF.

A global summary of cytokine fold‐change expression at both 4 and 16 h under shear is depicted in Figure 3b,c for both endothelial cell lines. Here, this plot represents the total number of cytokines that were either upregulated or downregulated at time points shared by the two endothelial cell types. For HUVECs under shear flow (Figure 3b), there is an approximate even distribution between those cytokine secretions that exhibited an increase (10 at 4 h; 8 at 16 h) and a decrease (11 at 4 h; 13 at 16 h) under shear. Conversely, the human microvasculature brain endothelial cells (Figure 3c) exhibited more increased cytokine production (17 at 4 h; 19 at 16 h), with relatively few factors in reduction (6 at 4 h; 4 at 16 h) under shear preconditioning. Indeed, it is perhaps useful here to examine the differences in the absolute value of cytokine concentration (pg mL^−1^) exhibited by these two human endothelial cell types. The absolute levels of six cytokines under shear (Figure 3e–f) highlight that, even among the cytokines in which both endothelial cell types secrete significantly more compared to static conditions (Figure 3a), HBEC‐5i endothelial cells produce these cytokines in vastly larger amounts, ranging from 2.2 to 36‐fold more than HUVEC under the same conditions. Of note, SDF‐1 and TNF‐B are exceptions here, with HUVEC showing 1.34 and 16.59‐fold higher concentration in comparison with HBEC‐5i at 4 h shear preconditioning. However, SDF‐1 and TNF‐B concentration increased rapidly in HBEC‐5i over time, reaching 2.58 and 2.22‐fold higher than HUVEC at 16 h shear flow, respectively. These observations suggest that brain microvascular endothelial cells not only have higher baseline levels of multiple inflammatory cytokines, but also demonstrate a more pronounced response to laminar shear stress by substantially enhancing the secretion of these key cytokines.

**Figure 4:**
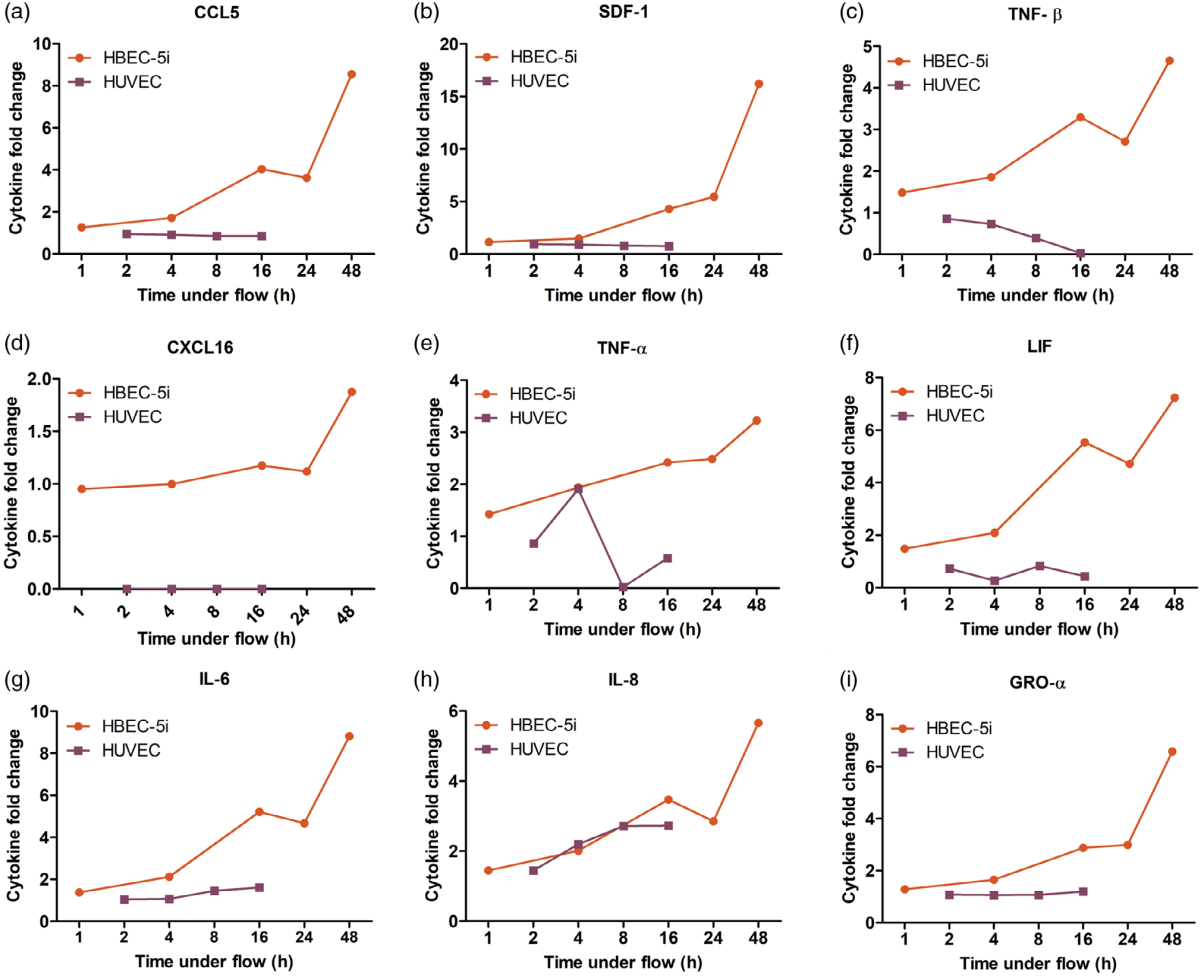
Shear‐mediated modulation of selected inflammatory factors in HUVEC and HBEC‐5i exhibit different temporal patterns. a–c) These plots illustrate the reverse pattern of cytokine secretion between HVEC and HBEC‐5i in response to shear flow preconditioning under the same condition. Over time, each cytokine in HBEC‐5i shows a significant upregulation up to 16‐fold at 48 h, whereas cytokine secretion is considerably downregulated in HUVEC. d–f) Analytes listed here are highly expressed in HBEC‐5i, with an increase in their secretion level under shear flow over the course of 48 h. However, the baseline level of these factors in HUVEC is extremely low, or completely absent in the case of CXCL16. g–i) The last row indicates a subset of chemokines with similar secretion patterns in HUVEC and HBEC‐5i under shear flow conditions, showing an increase in secretion levels over time.

One of the clearer results from this figure is the heterogeneity in shear stress‐mediated time‐dependent secretome expressed by the two endothelial cell types—further articulated in **Figure** **4**. Some signaling molecules were differentially expressed (e.g., CCL5, SDF‐1, TNF‐B were increased from HBEC‐5i up to 16.2‐fold but decreased from HUVEC up to 0.75‐fold; *p* < 0.001), with others, including TNF‐α, CXCL‐16, and LIF, expressed highly in HBEC‐5i (up to 7.2‐fold, *p* < 0.05), yet not at all from HUVEC (Figure 4d–f). A possible explanation for the observed differences in endothelial secretome changes in response to the 9 dyn cm^−2^ shear stress used here may be related to the magnitude of this shear stress in relation to its normal, physiological level. Indeed, endothelial exposure to high laminar shear stress (relative to its baseline) leads to suppression of proinflammatory pathways through the inactivation of NADH oxidase, whereas low shear stress exposure leads to the production of reactive oxygen species by activation of NADH oxidase, which subsequently leads to oxidative stress and promotes an inflammatory response.^[^ ^51^, ^52^ ^]^ The shear stress used here is slightly higher than the physiological shear range for HUVEC, which might explain the downregulation of most proinflammatory cytokines for this cell type. For HBEC‐5i, in contrast, it is actually in the lower range of the physiological shear for human brain capillaries, which may contribute to the activation of proinflammatory pathways.

Despite the observed differences in the effect of shear flow on the secretion level of most analytes from HBEC‐5i as compared to HUVEC, multiple factors including MCP‐1, PDGF, IL‐6, IL‐8, GRO‐α, EGF, and FGF‐2 showed a similar trend in both cell types (Figure 4g–i). These factors were significantly increased (up to 8.8‐fold; *p* < 0.001) from both endothelial cell types under flow throughout the time range analyzed (Figure 4c). Exposure to high laminar shear stress has been shown to activate the NF‐κB pathway, which is consistent with factors including PDGF, IL‐6, IL‐8, and MCP‐1.^[^ ^41^ ^]^ While other studies reported that laminar shear flow increases the level of growth factors such as FGF‐2 and EGF, we observed a decrease in the concentration of these factors after exposure to shear flow in both HBEC‐5i and HUVEC. This conflicting observation could be attributed to a difference in the exposure time to the shear stress, the magnitude of shear stress, and the cell type.^[^ ^41^ ^]^

**Figure 5:**
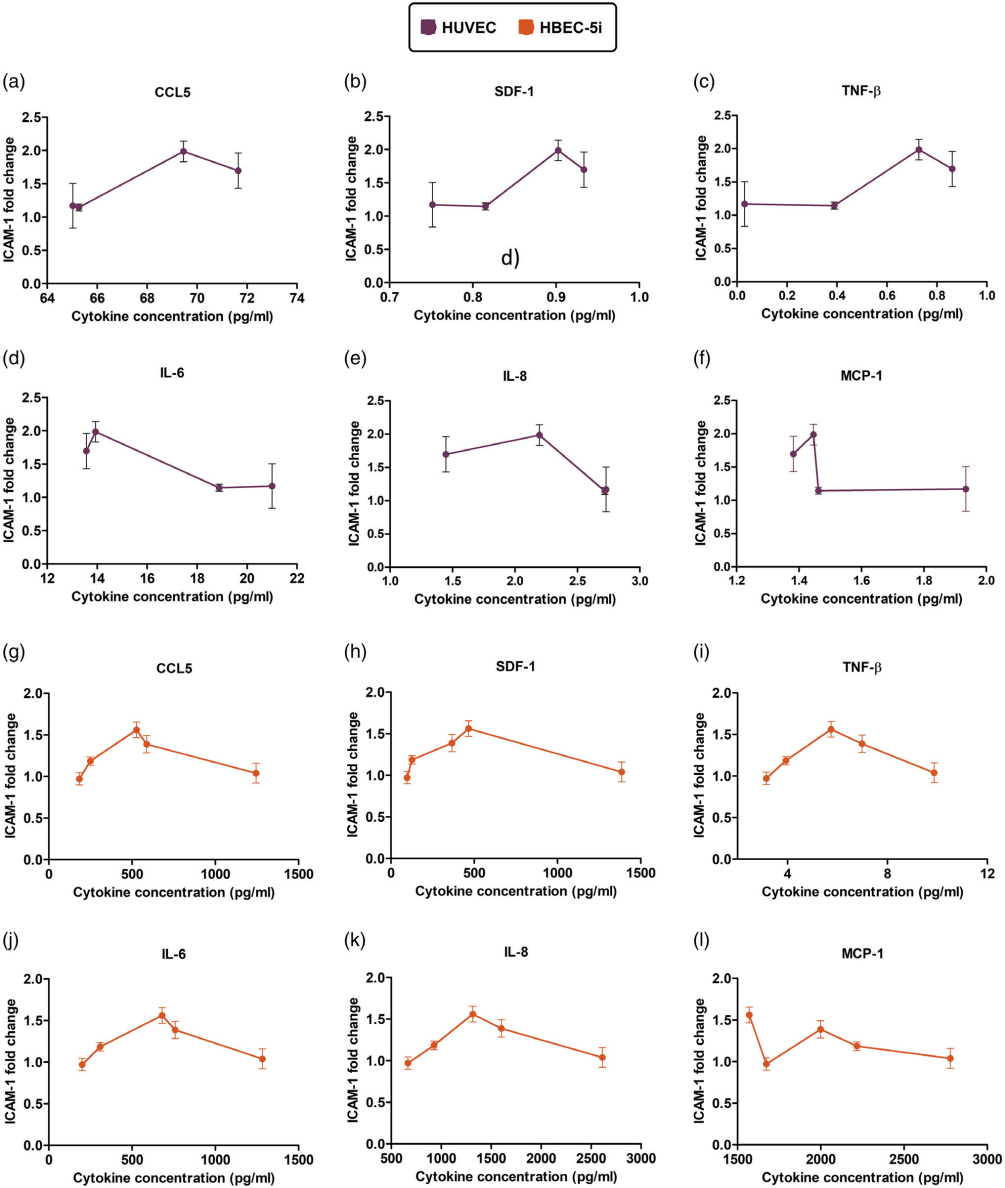
ICAM‐1 surface expression on endothelial cells is correlated with shear flow‐mediated cytokine secretion. These plots illustrate the correlation between individual cytokine concentration with ICAM‐1 fold change in either a–f) HUVEC or g–l) HBEC‐5i subjected to 9 dyn cm^−2^ laminar shear flow over time. The selected cytokines here are known to be involved in inflammation (CCL5, SDF‐1, TNF‐β) and chemotaxis (IL‐6, IL‐8, MCP‐1). As the plots show, ICAM‐1 surface expression in either HUVEC or HBEC‐5i has a nonlinear relation with inflammatory cytokines and chemokines concentration. This observation indicates that ICAM‐1 expression fold‐change transiently increases up to a certain concentration of each cytokine, followed by a decrease or reaching a plateau under higher concentrations.

As many of these cytokines have been shown to be involved in mediating inflammation and CAM expression, we sought to next correlate individual cytokine concentration with CAM expression, with a specific focus on ICAM‐1 for HUVEC and HBEC‐5i endothelial cells, shown here in **Figure** **5**. It has been shown that chemokine signaling transiently expedites the rolling and adhesion of immune cells by stimulating the generation of high avidity integrins within seconds of shear flow exposure, leading to firm adhesion of leukocytes to immunoglobulin family CAM such as ICAM‐1, VCAM‐1, and MAdCAM‐1 on the endothelial cells.^[^ ^53^ ^]^ This indicates that the upregulation of chemokines and the immunoglobulin family of adhesion molecules in an inflammatory condition probably takes place in close succession, both facilitating the firm adhesion of rolling immune cells to the endothelium.^[^ ^54^ ^]^ Regarding the direct influence of cytokines on the expression of ICAM‐1 in either HUVEC or brain endothelial cells, most studies focused on TNF‐α and IL‐1β, as it has been reported that they are the key mediators of CAM modulation.^[^ ^55^, ^56^ ^]^ O’Carroll et al.^[^ ^55^ ^]^ studied the effect of proinflammatory factors, specifically TNF‐α and IL‐1β, on the immunophenotype features of brain endothelial cells including the surface expression of ICAM‐1, revealing a differential response of brain endothelial cells to IL‐1β and TNF‐α. The treatment of brain endothelial cells with TNF‐α and IL‐1β (5 ng mL^−1^) triggered an inflammatory response, followed by a substantial increase in the secretion of 13 inflammatory factors including soluble ICAM‐1, soluble VCAM‐1, IL‐6, IL‐8, MCP‐1, and CCL5. They showed that the elevated levels of CCL5 and IL‐8 were associated with TNF‐α‐mediated inflammatory response. This is consistent with our results, observing a simultaneous increase in the secretion of TNF‐α, CCL5, and IL‐8 in HBEC‐5i under laminar flow, with the latter two possibly secondary to the enhanced level of TNF‐α. In contrast, the level of TNF‐α in HUVEC remained negligible over time, potentially explaining the downregulation of CCL5 and several other inflammatory cytokines in HUVEC. While IL‐8 secretion in HUVEC increased over time up to 2.7‐fold, this increase was less pronounced than HBEC‐5i, which showed 5.7‐fold enhancement in IL‐8 concentration under the same condition, likely attributed to the higher concentration of TNF‐α in the HBEC supernatant. Additionally, this study showed that conditioning of brain endothelial cells with a range of 50 pg mL^−1^ to 50 ng mL^−1^, TNF‐α and IL‐1β directly upregulated the surface expression of ICAM‐1 at 24 h posttreatment.^[^ ^55^ ^]^ Another study reported that the increased local concentration of chemokines considerably reduces the rolling distance of immune cells before their arrest at the site of inflammation, which confirms the supplementary role of chemokines and CAM in the adhesion of immune cells to the endothelium.^[^ ^57^ ^]^ Two examples are IL‐8 and MCP‐1 shown to stimulate the firm adhesion of CXCR1/CXCR2‐expressing neutrophils and CCR2‐expressing monocytes to the endothelium, respectively. As illustrated in Figure 5, the correlation of ICAM‐1 expression with IL‐8 and MCP‐1 under shear flow in HUVEC and HBEC‐5i follows a nonlinear pattern, with the peak ICAM‐1 expression happening at 4‐ and 24 h of shear flow preconditioning, whereas the IL‐8 and MCP‐1 secretion continue to increase over a time course of 48 h under flow condition. Theofilis et al.^[^ ^58^ ^]^ suggested that following the elevated level of inflammatory cytokines, critical CAM for rolling and adhesion of leukocytes, including ICAM‐1, VCAM‐1, E‐selectin, and P‐selectin will be upregulated on the endothelium, which is consistent with our results.

### 2.3 Ultrasound‐Assisted Treatment during Microbubble Perfusion

**Figure 6:**
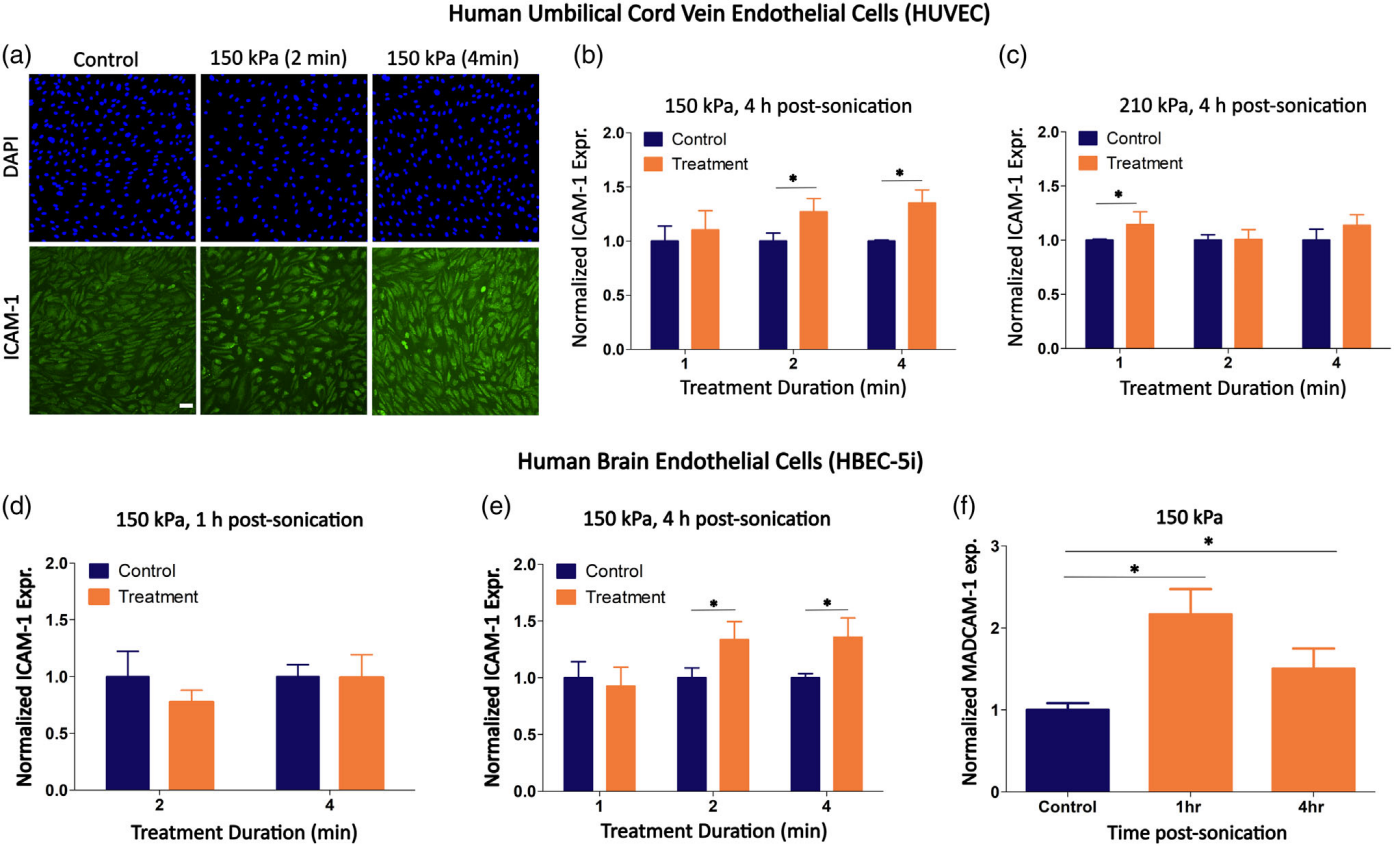
Ultrasound‐stimulated microbubbles increase CAM surface expression in both endothelial cell types. Endothelial cell‐seeded chamber slides were constantly perfused with microbubbles at a flow rate of 2.8 mL min^−1^ and sonicated with ultrasound at different acoustic pressures and sonication durations. a) Representative fluorescence microscopy examples of ICAM‐1 expression on HUVEC treated under different acoustic pressures and sonication durations. The surface expression of ICAM‐1 was assessed at 4 h post‐sonication. b,c) Quantitative analysis of ICAM‐1 expression from HUVEC demonstrated its upregulation under three ultrasound conditions: sonication at acoustic pressure of 150 kPa for 2 or 4 min, and 210 kPa for 1 min. d) For the brain endothelial cells (HBEC‐5i), quantification of ICAM‐1 surface expression at 1 h post‐sonication demonstrated no change in the expression as compared to the untreated cells. e) In contrast, ultrasound treatment under similar conditions significantly upregulated the surface expression of ICAM‐1 at 4 h post‐sonication, highlighting the importance of post‐sonication time in the modulation of ICAM‐1 expression for this endothelial cell type. f) Ultrasound treatment significantly increased the expression of MadCAM‐1 on HBEC‐5i as compared to untreated cells, with a peak expression observed at 1 h post‐sonication. The influence of ultrasound treatment was assessed on the expression of MadCAM‐1 on both HUVEC and HBEC‐5i, however, only HBEC‐5i experienced an upregulation of MadCAM‐1 in response to treatment. Therefore, the data for HUVEC are not shown here. No statistical difference between the treatment and sham control was observed unless marked on the plot.

**Figure 7:**
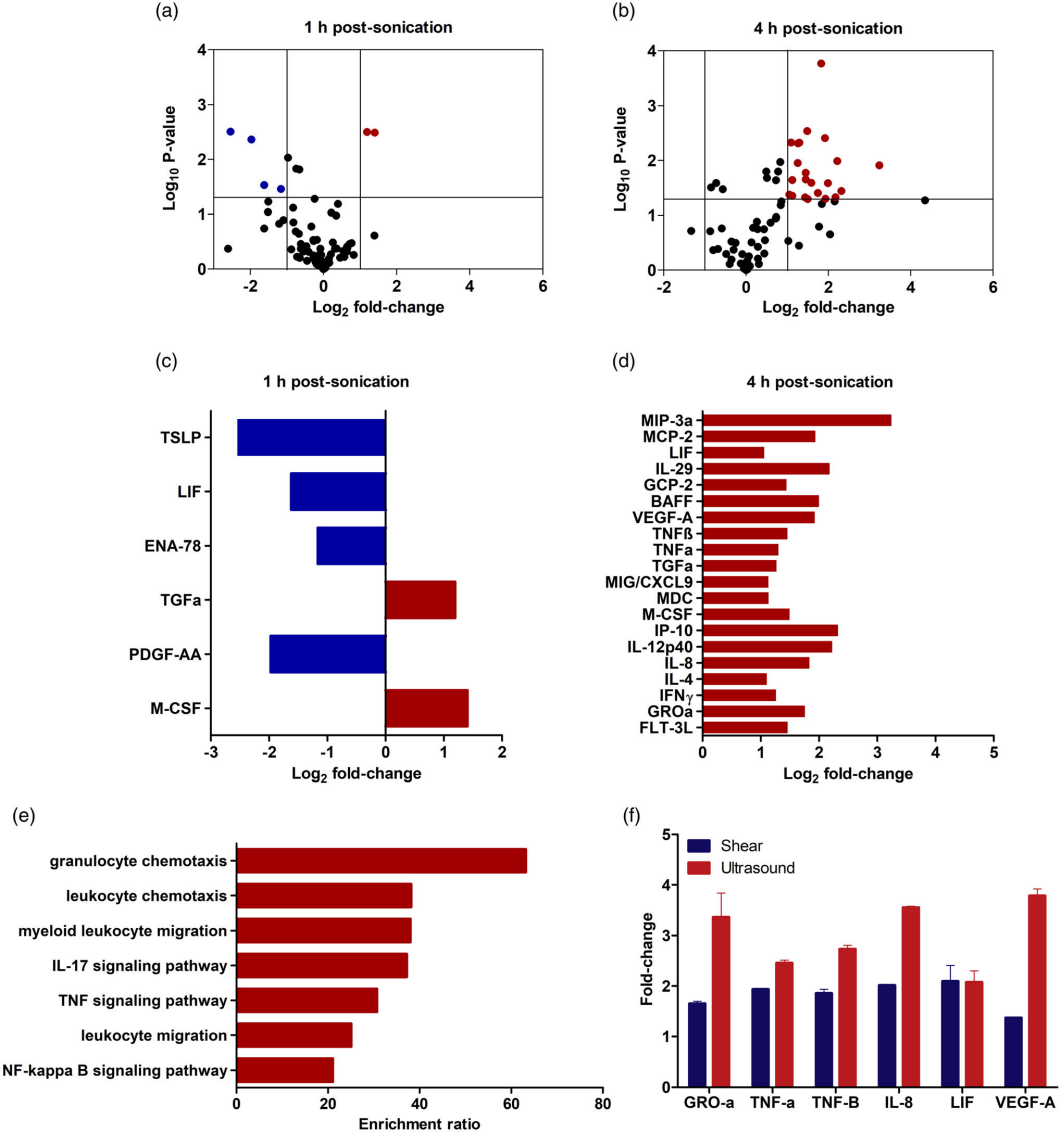
Microbubble‐mediated treatment modulates HBEC‐5i secretome profile. a,b) Volcano plots illustrating the differential secretion of various analytes at either 1 or 4 h after ultrasound treatment compared to the non‐sonicated cells. Each circle represents an analyte, where black ones show analytes with minimal or no change, red ones indicate analytes upregulated by at least twofold, and the blue circles represent analytes downregulated by at least twofold. c,d) Identification of cytokines with a minimum of twofold change and statistical significance at either 1 or 4 h after ultrasound treatment. Ultrasound treatment led to the differential secretion of a limited number of factors 1 h post‐sonication, whereas 4 h after treatment, 20 analytes experienced a significant upregulation, most of which are known for their immune‐activating function. e) Gene ORA of this 4 h dataset reveals that changes in the secretome are linked to known biological processes, including immune cell chemotaxis and migration, along with TNF and NF‐κB signaling. f) Comparison of the fold‐change secretion of seven cytokines 4 h after exposure to either shear flow (blue) or ultrasound‐stimulated microbubbles (red) in HBEC‐5i. The plot indicates that these cytokines exhibit a stronger response to ultrasound treatment than to laminar shear flow, likely due to higher shear stress exerted by microbubbles on endothelial cells.

With controlled shear‐flow modulation as a basis, we next treated human endothelial monolayers under flowing microbubbles with ultrasound; first using HUVECs. Employing the flow‐based data as a point of comparison, we compared ICAM‐1 surface expression at 1 and 4 h postsonication. Globally, we observed a modest increase in ICAM‐1 expression ranging from 10 to 30% when treated with any of the ultrasound conditions employed here compared to sham controls (**Figure** **6**a–c); with 150 kPa for 2 and 4 min, along with 210 kPa for 1 min obtaining statistical significance (*p* < 0.05 for all three of these conditions). Furthermore, the responses due to ultrasound treatment on HBEC‐5i were similar (Figure 6d,e), reaching increases of surface ICAM‐1 expression of 1.33‐fold and 1.36‐fold at the 150 kPa for 2 and 4 min treatments compared to untreated sham controls (*p* < 0.03; *p* < 0.02 respectively) at the 4 h postsonication time‐point. Indeed, our results here are consistent with studies that have explored the response of focused ultrasound and microbubbles on rodent brain vasculature. While direct comparisons are difficult due to the native acoustics and immunology differences between models, a subset of these in‐vivo studies note that focused ultrasound treatment to the brain can result in a 2.1‐fold increase in the number of ICAM‐1 expressing cells,^[^ ^59^ ^]^ a ≈2.5‐fold increase in ICAM‐1 gene expression,^[^ ^26^ ^]^ and an estimated 2.5‐fold increase in ICAM‐1 expression in brain tumors^[^ ^60^ ^]^ compared to nontreated regions, and depending on the specifics of the treatment paradigm used. Following this, we next explored the subset of CAM‐modulating acoustic conditions here on the expression of MadCAM‐1. While there were no significant differences determined from HUVECs (data not shown), we did observe a 2.16‐fold increase in surface MadCAM‐1 expression in HBEC‐5i under 150 kPa treatment for 2 min as compared to sham controls at 1 h postsonication (Figure 6f; *p* < 0.05); with this transient increase returning to baseline by 4 h post‐sonication. Indeed, plasma membrane properties including stiffness (which may be different between these two cells of endothelial origin) may play a role in ultrasound‐induced bioeffects under the same acoustic paradigm.^[^ ^61^ ^]^ Given the more robust response of the human brain microvascular cells, we next proceeded to examine the ultrasound‐modulated secretome of this cell type at both 1 and 4 h time points (see **Figure** **7**). In this figure, the first two panels depict volcano plots in which the −log10 *p*‐value is plotted against the magnitude of the fold‐change (in log_2_ units) of the ultrasound‐treated group relative to the untreated sham control. Here, each cytokine/analyte is denoted by a circle, with blue ones representing a strong (at least twofold; log_2_ of the fold change ≤−1) significant downregulation and red circles representing significant upregulation (log_2_ of the fold change ≥1) of a given cytokine. The identity of these analytes is shown in Figure 7c,d, with the earlier time point characterized by the decreased expression of 4 cytokines, while only 2 analytes demonstrated increased concentrations. At the later time‐point of 4 h, there is increased secretion of 20 cytokines, many of which serve immune‐activating roles (e.g., IL‐8, CXCL9, IP‐10, MIP‐3α). Beyond highlighting the fold‐change of individual secreted analyte concentrations, we performed gene overrepresentation analysis (ORA) of this 4 h dataset to ascertain whether and to what extent changes in the secretome can be linked to known biological processes (Figure 7e). Indeed, ORA analysis revealed multiple overrepresented pathways involved in immune cell recruitment. Specifically, enrichment ratios (ERs) were significant for several gene ontology (GO) terms including the chemotaxis and migration of leukocytes, and the modulation of IL‐17, TNF, and NFκB signaling pathways. We note here that these ultrasound‐induced changes are broadly consistent with whole‐tissue analysis performed on focused ultrasound‐treated preclinical models in the context of brain and brain tumors.^[^ ^26^, ^62^, ^63^, ^64^ ^]^ We acknowledge here that the role of these cytokines/chemokines is pleiotropic, highly contingent on the local environment, the presence of neighboring cells and tissue, and the magnitude and time course of its secretion. That being said, we explore the potential implications of ultrasound‐induced increases in selected secreted analytes reported here in the following sections.

Among the chemokines, most influenced by microbubble‐mediated focused ultrasound are shown here. IP‐10 (CXCL10) has been shown to strongly attract T‐cells and NK‐cells and has generally exhibited antitumor properties.^[^ ^65^ ^]^ In fact, CXCL10 immune therapy strategies have been successfully implemented in murine models of glioblastoma, particularly from a dendritic cell vaccine viewpoint,^[^ ^66^, ^67^, ^68^ ^]^ and very recently as an adjuvant therapy to immune checkpoint antibodies.^[^ ^69^ ^]^ Along with CXCL9—also shown here to increase under focused ultrasound treatment—CXCL10 secretion has shown a huge impact on the success of immune checkpoint inhibitor therapy.^[^ ^70^ ^]^ Given the relatively large increased secretion from human brain endothelial cells shown here (fivefold for CXCL10 and 2.2‐fold for CXCL9 over non‐ultrasound controls), these data suggest that focused ultrasound may aid in augmenting local T‐cell infiltration within the brain via the CXCL10 and CXCL9 axes.

Another analyte that was largely impacted by ultrasound treatment is VEGF‐A. Indeed, angiogenesis has long been considered one of the hallmarks of tumor progression,^[^ ^71^ ^]^ and VEGF is one of the most prominent and well‐studied angiogenic factors—with its expression positively correlating with tumor microvascular density and poor prognosis in many human tumors.^[^ ^72^, ^73^ ^]^ Numerous antiangiogenic agents targeted toward VEGF signaling are used clinically (e.g., bevacizumab targeting VEGF‐A; ramucirumab targeting VEGFR‐2)—however, the success of this approach has not met its full potential^[^ ^74^ ^]^—notably in glioblastoma, where despite its highly vascularized nature, anti‐VEGF treatment via bevacizumab corresponds with increased tumor cell infiltration (invasive potential)^[^ ^75^ ^]^ and has not translated to any improvement in patient survival. Indeed, among other tumor and angiogenesis‐related complexities,^[^ ^76^ ^]^ VEGF‐A can either positively or negatively regulate endothelial activation; with prolonged exposure inhibiting endothelial‐leukocyte interactions and decreasing endothelial responsiveness to proinflammatory cytokines. However, short‐term exposure has been shown to increase adhesion molecular expression and leukocyte infiltration in in‐vivo models.^[^ ^77^, ^78^ ^]^ Here, our results indicate increased levels of VEGF‐A up to ≈3.8‐fold sham‐treated control at 4 h post‐ultrasound exposure, which suggests that this may be used as a short‐term strategy to transiently increase leukocyte infiltration—although this requires further investigation.

Within the TME itself, it is well understood that the hostile physiological milieu—including the balance of pro and anti‐inflammatory molecules—plays a distinct role in hindering immunotherapies. To this end, particular attention has been paid to tumor‐associated macrophages (TAMs), which can make up a large portion (30–50%) of a solid tumor by weight.^[^ ^79^ ^]^ Indeed, TNF‐α plays a large role as a polarizing cytokine to induce TAMs toward the M1‐like macrophage phenotype; a subset of macrophages that strongly presents antigens and secretes many proinflammatory chemokines and cytokines, including TNF‐α itself.^[^ ^80^ ^]^ Furthermore, TNF‐α serves many other immune‐activating roles, including downregulating immune‐suppressing regulatory T cells within the TME,^[^ ^81^ ^]^ and attraction of neutrophils and monocytes to take part in antitumor responses^[^ ^82^ ^]^—notably when transiently present in high local concentrations.^[^ ^83^ ^]^

Our results also highlight that ultrasound‐assisted microbubble treatment increases endothelial secretion of IL‐8 (CXCL8), a potent chemoattractant for neutrophils. While traditionally not considered a major player in the TME, recent evidence suggests that neutrophils can play a role in tumor initiation, development, and progression. There are several studies that suggest neutrophils may exert direct cytotoxic activities or indirectly lead to tumor regression through the recruitment and induction of tumor‐specific T‐cell responses.^[^ ^84^, ^85^, ^86^ ^]^ Tumor‐associated neutrophils (TANs), similar to TAMs, can acquire an antitumor (N1) or immunosuppressive (N2) phenotype (e.g., in the absence/presence of TGF‐β), the extent to which can influence the outcomes of emerging immunotherapies, including immune checkpoint therapy (e.g., ^[^ ^87^ ^]^). Indeed, TANs differ from naïve bone‐marrow neutrophils in that they show an enhanced chemokine secretion profile,^[^ ^88^ ^]^ thereby contributing to a feedback mechanism for recruiting more neutrophils and other immune cells to the TME; specifically, chemokines including CCL3, CXCL2, and CXCL1 (GROα);^[^ ^89^ ^]^ some of which are also enhanced under the current ultrasound exposure regimen (Figure 7f).

Finally, we directly compare the modulated expression of seven cytokines that are shared between the shear‐flow mediated (Figure 4a) and ultrasound‐mediated therapy—see Figure 7f. Here, the fold‐change expression of LIF, GCP‐2, VEGF‐A, TNFβ, M‐CSF, IL‐8, and GROα exhibited by HBEC‐5i under shear flow (blue bars) and ultrasound and microbubble treatment (red bars) at 4 h is displayed. We note here that this is only a subset of those induced via flow (Figure 4a) and microbubble‐mediated ultrasound (Figure 7c,d). While both the flow‐induced ICAM‐1 surface expression and endothelial secretome modulation share some qualitative similarities with the ultrasound dataset, further research will involve in‐depth pathway analysis to highlight the degree of mechanistic similarity. Indeed, the shear stress induced by vibrating microbubbles is expected to be much larger in magnitude (≈15 kPa, or 15 kdyn cm^−2^)^[^ ^31^ ^]^ but over a much shorter timescale (≈μs—ms).

## 3 Conclusion

Here, we report on the endothelial overexpression of two surface adhesion molecules (ICAM‐1 and MadCAM‐1) and the modulation of endothelial secretome due to flow‐induced shear stress and microbubble‐mediated focused ultrasound therapy. Indeed, preconditioning under flow resulted in time‐dependent modulation of CAM expression, the character of which was dependent on the endothelial origin. Further, there was a heterogeneity in the time‐dependent modulation of cytokines and chemokines; with some significantly enhanced (up to 16.2‐fold; *p* < 0.001) from both endothelial cell types under flow (e.g., IL‐8, MCP‐1, MCP‐3), and others differentially expressed (e.g., CCL5, CXCL‐16, SDF‐1). Under ultrasound exposure, both endothelial cell types demonstrated increased surface levels of ICAM‐1 expression at 4 h postsonication, while only HBEC‐5i demonstrated enhancement of MadCAM‐1 compared to untreated controls. Microbubble‐mediated therapy resulted in large‐magnitude (more than twofold) and significant (*p* < 0.05) differential expression of 20 cytokines at 4 h post‐treatment, most of which had immune‐activating function and were within a similar subset to those induced by shear flow. Taken together, this dataset suggests that focused ultrasound‐induced changes in endothelial immunobiology share some, but not all, general characteristics of flow‐induced shear. Specifically, increased expression of immune‐modulating factors including surface CAM and proinflammatory secretions.

## 4 Experimental Section

4.1

4.1.1

##### General Cell Culturing

Two human endothelial cell models, either umbilical vein (HUVEC, C2519A; Lonza, Walkersville, MD, USA) or cerebral microvascular endothelial cells (HBEC‐5i, CRL‐3245TM; ATCC, Manassas, VA, USA), were employed in this study. The cerebral HBEC‐5i were seeded in precoated culture dishes with 1 mL gelatin (0.1%; ATCC) per 10 cm^2^, and were grown in DMEM:F12 (Wisent, Canada) cell culture medium supplemented with 10% fetal bovine serum (FBS, Wisent) and 40 μg mL^−1^ of endothelial growth supplement (ECGS, ATCC). Since HBEC‐5i is an immortal cell model (SV40 large T antigen transformation), they were used up to passage 20. HUVECs were cultured in endothelial cell growth medium (EGM‐2; CC3162, Lonza) up to passage 9. Ibidi chamber slides (Ibidi, Gräfelfing, Germany) were used for cell cultivation and treatment under flow. These slides have a 2.5 cm^2^ growth area, originally treated with collagen‐IV. However, 45 min prior to HBEC‐5i cultivation, the growth surface of the chamber slides was coated with 0.1% gelatin to provide extra support for HBEC‐5i adherence. Endothelial cells, either HBEC‐5i or HUVEC, were seeded in the chamber slides at a density of 120 000 cells cm^−2^. All seeded slides, whether cultured statically or under fluid flow conditions, were maintained within an incubator at 37 °C with 5% CO_2_% and 95% air atmosphere.

##### Flow Apparatus

The flow system has been described previously,^[^ ^37^ ^]^ see Figure 1a for experimental schematic. Briefly, the Ibidi flow system consists of a fluidic unit, air pressure pump, and flow controller software used to cultivate and treat endothelial cells under unidirectional laminar flow. To generate a constant flow, the fluidic unit was mounted with a yellow/green perfusion set with a total working volume of 13.3 mL. The air pump provided air pressure to drive the fluid flow through the fluidic unit. Additionally, the flow controller software was used for precise regulation of flow parameters including flow rate, shear stress, and flow cycle gradient. Before cell cultivation under flow, the perfusion set was primed with cell culture media and flow was applied for at least 30 min to remove any air bubbles in the fluid to prevent flow interruption. The Ibidi flow software calculates flow parameters based on the characteristics of the perfusion set, chamber slide geometry, and the viscosity of perfusion medium, which is assumed to be 0.0072 dyn cm^−2^ due to its similarity to that of water at 37 °C. Flow shear stress is determined via *τ* = *η* factor. Φ, where *τ* is shear stress (dyn cm^−2^), *η* is dynamical viscosity (dyn cm^−2^), and Φ is the flow rate of the perfused medium (mL min^−1^).

##### Cell Culturing under Shear Flow

To assess the effect of shear flow preconditioning on endothelial cell physiology, cells were cultivated statically in 0.2 mm μ–Slides for 2 h to allow cell adherence to the growth surface, followed by exposure to an augmented gradient of shear flow ranging from 1.5 to 7 dyn cm^−2^ for cell adaptation to the flow condition. Subsequently, cells were subjected to a shear flow of 9 dynes cm^−2^, corresponding to a 2.8 mL min^−1^ flow rate, for a duration ranging from 0 to 48 h. First, to assess the effect of time‐dependent shear flow on the endothelial secretome expressed by either HUVEC or HBEC‐5i, a small sample (100 μL) of cell media supernatant was collected from each chamber slide at different time points (from 1 to 48 h post‐gradient shear flow, depending on the endothelial cell type). Subsequently, the level of these factors was quantified using Luminex xMAP technology (see below). Additionally, HUVEC and HBEC‐5i cultivated under similar conditions were examined for the surface expression of either ICAM‐1 or MadCAM‐1 using immunohistochemistry (see below). The secretome and expression of ICAM‐1 and MadCAM‐1 under static cultivation were used as respective controls.

##### Immunohistochemistry

Endothelial cells were fixed with 2% paraformaldehyde (3C28557, Millipore Sigma) for 30 min, followed by incubation with 0.1% Triton X‐100 (Triton X‐100 Surfactant, Millipore Sigma) for cell permeabilization. To block the nonspecific binding of mABs and prevent the high‐intensity background, cells were incubated with a blocking buffer consisting of regular nonfat milk powder at a concentration of 0.05 g mL^−1^ in 5 phosphate‐buffer saline with 5% bovine serum albumin (PBS‐BSA) for 45 min. Subsequently, cells were incubated with either ICAM‐1 monoclonal antibody (MA5407, Invitrogen) or MadCAM‐1 polyclonal antibody (PA598417, Invitrogen) for 1 h, followed by washing five times with PBS‐BSA to remove any unbound antibodies. Due to the specific geometry of chamber slides which can trap air bubbles if medium is fully removed, continuous medium exchange was used for washing with PBS‐BSA. For each wash, 50 μL PBS‐BSA was added through one inlet and removed from the opposite inlet. This process was repeated three times for each wash, resulting in a total of 15 times to complete the 5 washes with a total volume of 750 μL PBS‐BSA per slide. Next, the cells were incubated with secondary antibodies including either Alexa Fluor 488 (Donkey anti‐mouse IgG, A21202, Invitrogen) or Alexa Fluor 555 (goat anti‐rabbit IgG, A21428, Invitrogen), respectively, and washed five times with PBS‐BSA. At the end of this process, cell nuclei were counterstained with DAPI (Thermofisher) for 2 min. As a negative control, some slides were stained with only primary or secondary antibodies. All the staining steps were performed at room temperature. To examine the surface expression of CAMs, cells were visualized using an inverted fluorescence microscope (Nikon Ti2A, Melville, NY) equipped with an LED light source (D‐LEDI, 10–50% intensity, Nikon) and DAPI (#96389, 378/447 nm; Nikon), GFP (#96392, 466/525 nm; Nikon), and DAPI (#96394, 554/609 nm; Nikon) filter cubes. At least five nonoverlapping locations (1.2 by 1.2 mm field of view) from each slide were imaged using a 10× objective (#MRH00101, Nikon), and images were quantified using in‐house MATLAB software to calculate the signal intensity per cell.

##### Multiplex Cytokine Release Assay

For the assessment of human cytokines, chemokines, and growth factors under either shear flow preconditioning or ultrasound treatment, multiplex immunoassay was used to measure the secretion concentration of 96 factors in each sample. The multiplex analysis utilizes uniquely color‐coded magnetic beads, each of which is coupled with a specific capture antibody for each analyte. To be able to examine the level of 96 factors, two 48‐plex kits (MilliporeSigma, Burlington, Massachusetts, USA) were used: Panel A consist of sCD40L, EGF, Eotaxin, FGF‐2, FLT‐3 Ligand, Fractalkine, G‐CSF, GM‐CSF, GROα, IFN‐α2, IFN‐γ, IL‐1α, IL‐1β, IL‐1RA, IL‐2, IL‐3, IL‐4, IL‐5, IL‐6, IL‐7, IL‐8, IL‐9, IL‐10, IL‐12(p40), IL‐12(p70), IL‐13, IL‐15, IL‐17A, IL‐17E/IL‐25, IL‐17F, IL‐18, IL‐22, IL‐27, IP‐10, MCP‐1, MCP‐3, M‐CSF, MDC, MIG/CXCL9, MIP‐1α, MIP‐1β, PDGF‐AA, PDGF‐AB/BB, RANTES, TGFα, TNF‐α, TNF‐β, and VEGF‐A, and panel B consists of 6CKine, APRIL, BAFF, BCA‐1, CCL28, CTACK, CXCL16, ENA‐78, Eotaxin‐2, Eotaxin‐3, GCP‐2, Granzyme A, Granzyme B, HMGB1, I‐309, I‐TAC, IFNβ, IFNω, IL‐11, IL‐16, IL‐20, IL‐21, IL‐23, IL‐24, IL‐28A, IL‐29, IL‐31, IL‐33, IL‐34, IL‐35, LIF, Lymphotactin, MCP‐2, MCP‐4, MIP‐1δ, MIP‐3α, MIP‐3β, MPIF‐1, Perforin, sCD137, SCF, SDF‐1, sFAS, sFASL, TARC, TPO, TRAIL, and TSLP. First, antibody‐coated microspheres are incubated with the sampled cell supernatant, followed by washing to remove the nonattached analytes. Next, the coated beads with the captured analytes are incubated with biotinylated antibodies against all the targets, followed by another wash. At the end, the beads are exposed to the reporter streptavidin‐phycoerythrin to detect the bound analytes. Luminex 200 (Luminex, Austin, TX, USA), which is a dual laser system, was used to identify the detected analytes as well as their concentration. In this system, the first laser excites the internal color of each bead to identify the presence of each analyte, whereas the second laser quantifies the intensity of the phycoerythrin signal, which is proportional to the concentration of each analyte. For a subset of this dataset, gene ORA was performed using web‐based software (https://www.webgestalt.org/). This was to determine statistically significant (Fisher's exact test) over‐representation of the modified secretome set compared to the human protein‐encoding genome. The ER reported is defined here as the number of genes within our list that fall within a given functional/signaling category divided by the expected number, given the total number of genes in the database (KEGG database) for that category. Only those with false discovery rates (adjusted for multiple comparisons) less than 0.05 are shown.

##### Ultrasound‐Assisted Treatment during Microbubble Perfusion

A customized acoustically coupled inverted microscopy system was developed by aligning a coupling cone for the transducer to the microscope field of view to visualize the ultrasound bioeffects during the sonication. The coupling cone was fixed at 27 mm distance from the growth area of the chamber slides, with 45° angle from the normal to minimize ultrasound reflection. Given the geometry of the system used here, the acoustic beam width projected along the surface of the cells is ≈3.5 mm. Acoustic characterization of the beam resulted in a transmission loss due to the chamber slide on the order of 5%.^[^ ^37^ ^]^ To enable treatment under flow, a fluidic system was placed next to the ultrasound delivery system and mounted with a perfusion set. The reservoirs were primed with 15 mL cell culture medium, and the fluid flow was initiated using the Ibidi pump system for 30 min to remove any air bubbles in the fluid. Next, a cell‐seeded chamber slide was connected to the perfusion set and perfused with Definity microbubbles (1:2500 dilution) for 2 min to allow a uniform distribution of microbubbles. Definity microbubbles were activated from room temperature using the Vialmix shaker for 45 s, after which they were allowed to rest for another 15 min to equilibrate to room temperature.^[^ ^90^ ^]^ Native, undiluted microbubble size and concentration were confirmed using a Coulter Counter Multisizer 4 (Beckman Coulter, Indianapolis, IN, USA) to be consistent with the literature (≈10^10^ bubbles mL^−1^; bimodal volumetric peak in size distribution). The surface of the slide was water coupled with the coupling cone, and four nonoverlapping regions from the slide were sonicated (1 MHz frequency, 20 cycles, 1 ms PRI) at a peak‐negative pressure of either 150 or 210 kPa, with a sonication duration ranging from 1 to 4 min. The acoustic beam was characterized in a separate water‐tank with a hydrophone (HGL‐200, Onda Corp., Sunnyvale, USA). The surface expression of either ICAM‐1 or MAdCAM‐1 was assessed as a factor of time post‐sonication, either 1 or 4 h. For a subset of these, the supernatant was collected for cytokine analysis. In this case, immediately after ultrasound treatment, the flow was stopped, and the chamber slide was disconnected from the fluidic unit to prevent the dilution of cell supernatant with the media in the reservoirs. To be consistent with all other experiments, the chamber slides were placed in an incubator at 37 °C with 5% CO_2_ for either 1 or 4 h. Next, 100 μL of cell media supernatant was sampled from each slide, and the concentration of 96 factors, similar to the shear‐flow assay, was examined using the same multiplex immunoassay.

##### Justification of Parameter Selection

It is worth discussing the justification for the shear flow, molecular biology readouts, and acoustic conditions employed in this study. First, the shear stress in arterial vessels and veins has been reported to range between 10–70 and 1–6 dyn cm^−2^, respectively,^[^ ^91^ ^]^ whereas brain microvasculature experiences shear flow ranging from 5 to 23 dyn cm^−2^.^[^ ^51^ ^]^ Our selected shear stress of 9 dyn cm^−2^ falls on the lower end of these physiological ranges, albeit slightly above the reported range for venous blood flow. In terms of our selection of endothelial surface expression markers, downregulation of ICAM‐1 and MadCAM‐1 (and others) have been reported in tumor‐associated vessels from a range of human malignancies.^[^ ^6^, ^92^ ^]^ First, ICAM‐1 is a notably important adhesion molecule in the context of immune cell adhesion and trafficking and has long been studied in this context.^[^ ^93^, ^94^ ^]^ Further, MadCAM‐1 is expressed on human brain endothelium and plays a role in NK cell transmigration.^[^ ^95^, ^96^ ^]^ With respect to the ultrasound treatments, the microbubble concentration was chosen (1:2500 dilution) to fall within the clinical implementation, which typically reaches a dilution of ≈1:2500–1:5000 after intravenous administration. The ultrasound parameters were chosen after a pilot study to minimize losses in cell viability and detachment in both endothelial cell lines, while still within the range of those reported for immunomodulation studies elsewhere, and relevant to therapeutic parameters used clinically.

##### Statistical Analysis

The data in this study have been analyzed using GraphPad Prism and are reported as mean ± SD. To assess the surface expression of CAMs, a minimum of 4 independent slides were analyzed, and imaging was performed on 5–10 randomly selected regions on each slide with at least *n* = 3 slides per condition. The level of CAM expression was quantified using in‐house software in MATLAB by measuring the fluorescence intensity of the cells. All secretome data were assessed on at least *n* = 2 samples per condition. Statistical comparison across different treatment regimens on the CAM expression was made by two‐tailed, unpaired two‐sample Student's *t*‐tests. A *p*‐value of <0.05 was taken to be statistically significant.

## Conflict of Interests

The authors declare no conflict of interest.

## Author Contributions

**Elahe Memari**: conceptualization: (equal); data curation: (lead); formal analysis: (equal); investigation: (lead); methodology: (equal); writing—original draft: (lead); writing—review & editing: (equal). **Davindra Singh**: data curation: (supporting); methodology: (supporting); writing—review & editing: (supporting). **Ryan Alkins**: conceptualization: (supporting); writing—review & editing: (supporting). **Brandon Helfield**: conceptualization: (equal); formal analysis: (equal); funding acquisition: (lead); investigation: (equal); methodology: (equal); supervision: (lead); writing—original draft: (equal); writing—review & editing: (equal).

## Data Availability

The data that support the findings of this study are available from the corresponding author upon reasonable request.
